# Determining the Mean Dermatology Life Quality Index of Skin of Color (SOC) Patients With Vitiligo: A South Asian Perspective

**DOI:** 10.7759/cureus.70709

**Published:** 2024-10-02

**Authors:** Aqsa Habib, Hina Aslam, Anjum Muhammad, Arjun Mahajan, Sohail Muhammad, Rami J Ghafar, Burhanuddin Patelwala, Umair Ajmal, Yousra Abdul Khaliq, Rashid Z Ali, Gurnam Virdi

**Affiliations:** 1 Dermatology, Pak Emirates Military Hospital, Rawalpindi, PAK; 2 Pharmacology, King Edward Medical University Lahore, Lahore, PAK; 3 Dermatology, Harvard Medical School, Boston, USA; 4 Dermatology, Mian Rashid Hussain Shaheed Hospital, Nowshera, PAK; 5 Internal Medicine, University Hospitals Bristol and Weston NHS Foundation Trust, Bristol, GBR; 6 Internal Medicine, Sligo University Hospital, Sligo, IRL; 7 Radiology, Pakistan Institute of Medical Sciences, Islambad, PAK; 8 Dermatology, Sandeman Provincial Hospital, Quetta, PAK; 9 Surgery, Pak Emirates Military Hospital, Rawalpindi, PAK; 10 Dermatology, Academy of Aesthetic Medicine, Shipley, GBR

**Keywords:** dermatology life quality index, skin of color, skin of color dermatology, vitiligo, vitiligo dlqi, vitiligo extent score, vitiligo severity

## Abstract

Background: Vitiligo has a special significance to the skin of colour (SOC) population because depigmentation is more obvious in the SOC population. As a result, vitiligo exerts a huge stigma on the affected individuals. The literature so far has reported on the health-related quality of life (QOL), including the dermatology life quality index (DLQI), and has demonstrated the profound effect of vitiligo on a patient’s QOL. Those affected by vitiligo are found to have lower self-esteem when compared to the non-affected individuals. The impact on the SOC population is even graver and underrepresented. Thus, the objective of this study was to report the mean DLQI in vitiligo in SOC individuals.

Materials and methods: This descriptive, cross-sectional study was performed in the dermatology outpatient department of a tertiary healthcare setup in Rawalpindi Pakistan. A total of 113 patients suffering from vitiligo, aged 15 to 65 years, both male and female, were included in the study. In all the cases, the DLQI score was noted.

Results: With the age ranging from 15 to 65 years and a mean age of 34.96 ± 9.59 years, 70% (n=77) of the patients were between 15 to 40 years of age. The male-to-female ratio was 1:1.3, with male patients comprising 38.21% (n=47) and females being 61.79% (n=66). The mean DLQI score of the vitiligo patients in our cohort was 9.39 ± 6.35.

Conclusion: This study concluded that the mean DLQI score was higher in female patients and in vitiligo universalis patients, compared to other variants of vitiligo. The SOC population affected with vitiligo is at a higher risk of having a decreased QOL and hence may need special attention with regard to quality health delivery services.

## Introduction

Vitiligo is a well-noted, acquired idiopathic dermatological ailment presenting with depigmented skin lesions as a result of circumscribed, progressive, hypo-melanosis of the skin and integumentary system. With a worldwide occurrence, the incidence of vitiligo is reported to be 0.5-2.0% [1]. Although the disease may be disfiguring in all races, its negative impact on the skin of colour (SOC) population is an important and large population, lying on the darker end of the Fitz-Patrick scale of skin colour, may be particularly higher as a result of the enhanced contrast of the lesions. Vitiligo may exert a host of significant psychosocial sequelae, carrying a social stigma and a resultant fall in the quality of life (QOL), particularly when vitiligo lesions present over the exposed sites of the body such as the face. In such cases, there is a high chance that the quality of personal and social life will be significantly affected in a negative way with the patient’s perception playing an important role in it [2].

For the SOC population afflicted with vitiligo, the impact may be highly significant as depigmentation remains obvious on darker skin, imparting an enormous stigma to the disease [2]. Several researches have shown a significantly negative impact on the mean Dermatology Life Quality Index (DLQI) affected by vitiligo [1,3-5]. Patients with vitiligo are reported to have a decreased self-reported self-esteem score as compared to the non-affected controls and researchers have reported a higher incidence of significant psychosocial co-morbidity (anxiety, depression and adjustment issues) in as much as 25% of individuals suffering from vitiligo [6].

It is pertinent to mention that despite being a largely asymptomatic disorder, the effects of vitiligo on QOL are far more related to psychological issues, namely a decrease in the level of self-confidence, unsuccessful or struggling social relationships, the feeling of unpleasant body images, and a fall in the quality of marital relations, than exclusively to physical aspects. It has been demonstrated in previous research that individuals suffering from vitiligo possess a higher risk of social stigmas, discrimination and challenges about lifestyle modification due to their disease [7]. The SOC population, particularly those residing in Western countries like the United Kingdom and the United States are at risk of developing such psychosocial comorbidities due to their comparatively limited access to healthcare facilities and the treatment modalities not tailored according to their specific needs.

Therefore, this study is designed to determine the mean DLQI score in patients suffering from vitiligo in a Pakistani cohort from Rawalpindi, a city where the majority of the population is SOC, using a simple, user-friendly, validated DLQI questionnaire, since the local data is sparse on this important aspect of this skin disorder. The results can emphasize and report the burden of the disease as well as the proper evaluation of QOL in this particular population so that early treatment for vitiligo with multiple treatment strategies could be undertaken to improve the overall QOL and decrease morbidity.

## Materials and methods

This was a descriptive cross-sectional study conducted at the outpatient dermatology department of the Pak Emirates Military Hospital, Rawalpindi, Pakistan, following due approval of the Ethical Committee Pak Emirates Military Hospital (approval number: A/28/22/EC/495/2022). The study was conducted over six months.

Eligibility criteria

Patients of all genders suffering from vitiligo with ages ranging from 15 to 65 years were included. The diagnosis of vitiligo was made on clinical and Wood's lamp examination to determine the characteristic chalky white accentuation of the lesions present in vitiligo. The exclusion criteria comprised any patient with a personal or family history of substance abuse, mental illness, and other obvious reasons for depression including chronic medical and surgical ailments. This approach was employed to avoid any bias and prevent other conditions that directly or directly affect the quality of life in the patients. Patients with non-characteristic hypopigmented lesions or equivocal findings of Wood's lamp examination were also excluded from the study.

Sample calculation

Employing non-probability, consecutive sampling with a 95% confidence level and 0.01 absolute precision and taking the DLQI of the vitiligo patients as 5.2 ± 5.4, the sample size was calculated [8]. Accordingly, a total of 113 patients were included in the study after obtaining explicit informed and written consent from them.

Tool

The DLQI aims to assess and measure the health-related QOL of adult patients afflicted with a dermatological ailment. Following the standard questionnaire, 10 questions were asked of each participant by pre-trained dermatologists in the language easily comprehended by the volunteers. The questions were classified into six headings items: (i) Symptoms and feelings such as pain, stinging, soreness and feeling of embarrassment/self-confidence (questions 1 and 2, respectively), (ii) Daily activities of the patient such as shopping and looking after one’s home or garden or the choice of the clothes (questions 3 and 4, respectively), (iii) Leisure activities such as hobbies, sports (questions 5 and 6, respectively), and (iv) personal relationships especially related to work or study and relationship with close friends, relatives, and sexual difficulties (questions 8 and 9, respectively), each component with maximum score of 6, and (v) Work and school (question 7), and (vi) Negative effects of treatment (question 10), each with a maximum score of 3 (as shown in detail in the Appendices) [9]. The DLQI score was calculated by adding up the score of each question with a minimum score of 0 and a maximum score of 30. A higher value of DLQI implied a corresponding higher impact on the QOL. Age, gender, duration of disease, place of living (rural/urban), family history and type of vitiligo were also recorded.

Data analysis

The data analysis was carried out using IBM SPSS Statistics for Windows, Version 25.0 (Released 2017; IBM Corp., Armonk, New York, Unitred States). The age, duration of the disease, and DLQI score were presented as mean and standard deviation. Effect modifiers like age, gender, duration of disease, place of living, family history of vitiligo and type of vitiligo (vitiligo vulgaris/vitiligo universalis/focal/segmental/acrofacial) were controlled via stratification and post-stratification independent ‘t’ test was employed. A p-value of <0.005 was considered as significant.

## Results

Of the 113 patients, 66 (61.79%) were female while 47 (38.21%) were male. The ratio of the two genders was 1:1.3. While the age range was 15-65 years, the mean age was 34.96 ± 9.59 years. The majority of the patients (n=77; 68.14%) were between 15 and 40 years of age. The mean duration of vitiligo was 4.02 ± 1.08 years with around 75% having less than four years’ duration, while the rest suffered for more than four years. More than half (57.5%) of the patients were rural residents while the rest lived in urban settings. Similarly, 51 (42%) reported a positive family history of the disease. The mean DLQI score of the vitiligo patients was 9.39 ± 6.35 with a significant difference (p-value 0.04) noted in DLQI scores with respect to gender, where females showed a higher DLQI score than male patients, as shown in Table 1.

**Table 1:** Stratification of DLQI scores with regard to gender (N=113) Effect modifiers like gender (and other variables) were controlled via stratification and a post-stratification independent ‘t’ test was employed. A p-value of <0.005 was considered as significant DLQI: Dermatology Life Quality Index

Gender	DLQI score	p-value
	Mean±SD	0.044
Male	6.38±6.51	
Female	10.81±5.90	

The distribution of individuals as per the type of vitiligo is shown in Table 2, where segmental vitiligo was found to be the commonest reported variant in 40 (35%) patients followed by vitiligo vulgaris in 36 (31%) patients. No significant difference in DLQI (p-value 0.09) between the two age groups of 15-40 years and 41-65 years was noted. The duration of disease and urban/rural settings, family history, type of vitiligo, and presence or absence of diabetes mellitus or other comorbidities did not seem to affect the DLQI scores in our cohort. All of these variables are tabulated in Table 3.

**Table 2:** Distribution of patients with regard to the type of vitiligo (N=113)

Type of Vitiligo	Frequency	Percentage
Vitiligo vulgaris	36	31.86%
Vitiligo universalis	11	9.73%
Focal vitiligo	19	16.81%
Segmental vitiligo	40	35.40%
Acrofacial vitiligo	07	6.19%

**Table 3:** Stratification of the patients with respect to different variables (N=113)

Parameter		Frequency	Percentage
Age	< 40 years	77	68.14%
	> 40 years	36	31.86%
Gender	Male	47	38.21%
	Female	66	61.78%
Duration	≤4 years	83	73.5%
	≥4 years	30	26.55%
Living Background	Urban	48	42.48%
	Rural	65	57.52%
Family History	Yes	62	58.54%
	No	51	41.46%

## Discussion

Vitiligo, like many dermatological ailments, can have a significant adverse outcome on individual self-image and psychological well-being [10,11]. It is not uncommon for vitiligo patients to have feelings of distress and stigma due to their appearance. Most vitiligo patients have been found to have feelings of embarrassment, that can bring about a fall in self-esteem and a resultant social isolation [6].

The DLQI of vitiligo was considerably higher in our observation when compared to a previous observation by Morales et al. which observed a DLQI of 5.2±5.4 [8]. The probable reason for this could be the reason that their study was carried out on a cohort that was ethno-geographically different from ours. The same explanation could be applied to the observations of Wang et al. on the Asian population [12], which reported a lower DLQI of 4.8±3.6, contrasting with our findings. However, observations identical to ours were made by various Indian studies like Parsad et al. [11] and Sangma et al. [13], who reported increased DLQI scores (10.67 and 9.08±4.46, respectively) in their patients. Similar observations were reported in Saudi Arabia by Al-Robaee [14] and Al-Mubarak et al. [15]. These significant findings may be explained by the fact that Indians and Pakistanis from the subcontinent and Arabs in the Middle East, lie on a similar darker part of the Fitzpatrick skin spectrum than those in Europe, the Mediterranean, and Southeast Asia.

Our study reports that, among volunteers who had higher DLQI scores, female patients represented three times more than males (male/female ratio = 1: 2.98), a finding also reported by Akrem et al. [16] and Al-Mubarak et al. [15] in the recent past. Our study, thus, augments the notion that the detrimental effect of vitiligo on the female population is far more than their male counterparts, an observation that is in contrast to previous findings by Parsad et al. [11], who reported that DLQI in vitiligo is not adversely affected by the patient’s gender. This statistically significant gender difference in DLQI in our study implies the need for more attention to female patients in terms of health delivery services and psychosocial support pertaining to vitiligo. When seen from a larger perspective, the higher DLQI score in the female population also points to the gender gap with regard to poorer socioeconomic security and access to healthcare services in the female population in the Indo-Pak subcontinent. This and the general fact that women specifically face more humiliation and self-consciousness about vitiligo, affecting their choice of clothing and daily routine, is objectively reported by our study.

According to our study, vitiligo brings about a similar adverse effect on patients' QOL and self-perception similar to other dermatological ailments like psoriasis [17] and eczema [18]; however, the effects by other pigmentary conditions such as melasma were found to be far lower in various South Asian studies [19,20], implicating that skin ailments causing depigmentation impart higher morbidity than their pigmentary counterparts in the SOC population.

Parsad et al. [11] and Hedayat et al. [21] reported a highly significant relationship between the duration of vitiligo and its negative effect on the DLQI scores. The current study suggests that the lesions on the visible/exposed part of the skin score higher compared with the lesions on covered body sites in the DLQI. Similarly, individuals suffering from vitiligo lesions on the exposed parts of the body were reported to have faced a higher level of stigmatization, a finding that was in line with observations reported by Schmid-Ott et al. [22].

The highest mean DLQI score (13.27) was observed in patients suffering from vitiligo universalis, as compared to localized disease with a statistically significant p-value of < 0.001. This observation was consistent with a report from Saudi Arabia [14]. Thus, we report that a higher DLQI score is consistent with a high activity of the disease, the presence of lesions on the exposed parts of the skin, and a higher involvement of the body surface area in the SOC population. These factors can invariably contribute to an increase in disease-related stress levels and hence potentially affect the patient's response to the therapeutic strategies in a negative way. Our observations of vitiligo in the SOC population are supported by an earlier South Asian study which observed that vitiligo patients with low DLQI scores responded far better to treatment than those with higher DLQI scores [11].

Bhuptani et al. reported the mean DLQI score to be 6.14 in their cohort [7]. While their research observed a statistically significant difference in the values of the mean DLQI scores of married vs unmarried individuals, no other statistically significant difference was found in any further groups/variables, a finding consistent with our study. The majority of the previous studies have reported a marked difference in the mean DLQI scores. As compared to our study, Al-Robaee [14] and Al-Mubarak et al. [15] had very high mean DLQI while Chahar et al. [5] and Sangma et al. [13] had similar mean DLQI (as shown in Table 4). Conversely, studies by Kent et al. [23] and Morales-Sánchez MA et al. [8] reported far lower DLQI values in their cohorts. This implies that the impact of vitiligo on psychosocial health is multifactorial with social, cultural, and economic elements exerting a significant impact on DLQI in vitiligo.

**Table 4:** Comparison of DLQI scores as reported by earlier studies compared to the current study DLQI: Dermatology Life Quality Index

Studies in the literature	DLQI score (mean±SD)
Chahar et al., 2018 [5]	8.64±4.32
Morales-Sánchez et al., 2017 [8]	5.2±5.4
Sangma et al., 2015 [13]	9.08±4.46
Kent et al., 1996 [23]	4.8±2.32
Al-Robaee, 2007 [14]	14.7±6.67
Al-Mubarak et al., 2011 [15]	17.1±6.72
Bhuptani et al., 2020 [7]	6.14±1.34
Present study	9.39±6.35

Limitations of the study

The study had several limitations. First, the sample size was small. Second, being an observational cross-sectional study, we were unable to assess the DLQI following any intervention. Third, despite being calculated by trained dermatologists, there remains a possibility of inter-observer bias. Fourth, since a large population of the cohort belongs to a low socioeconomic background, it is possible that the patient perceptions, answers, and the resultant DLQI may have been affected by the various socioeconomic challenges that are prevalent in a country like Pakistan.

## Conclusions

In our study conducted on a primarily SOC population, it was found that vitiligo has a high impact on the DLQI of affected individuals, with an increased score in female patients and those suffering from the vitiligo universalis variant of the disease. A proper evaluation of the QOL in these individuals is highly recommended to ensure better therapeutic outcomes. Similarly, earlier employment of combination treatment strategies for vitiligo patients should be carried out to bring faster and earlier improvement to their QOL and decrease the associated morbidity.

## Figures and Tables

**Figure 1:**
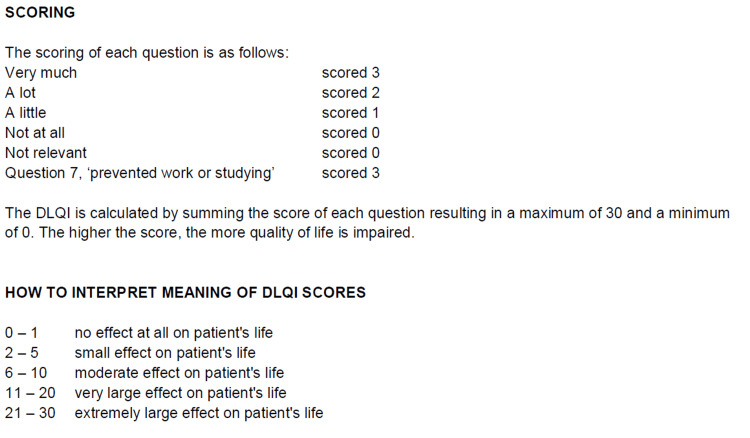
Calculation of DLQI and its interpretation. DLQI: Dermatology Life Quality Index

**Figure 2:**
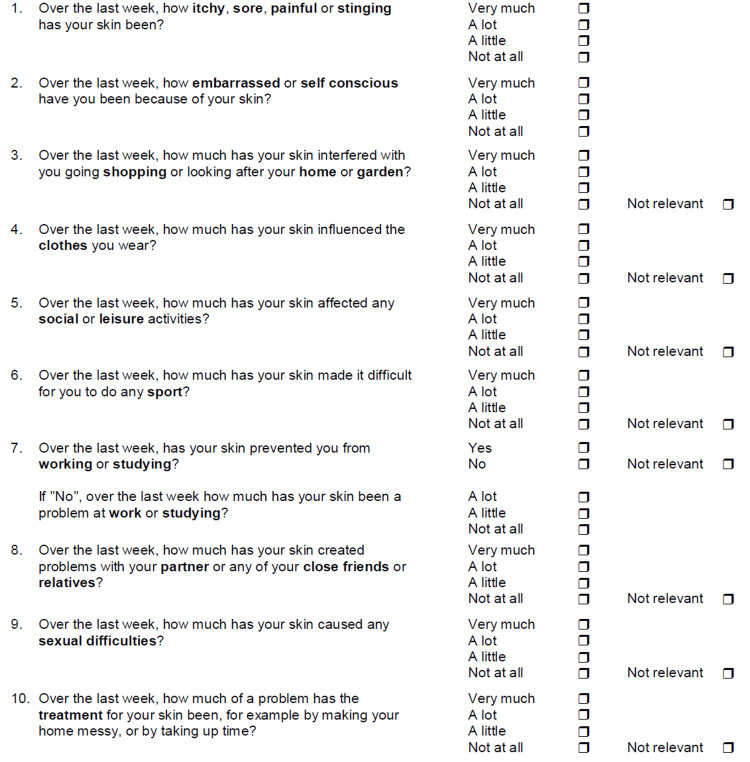
Questionaire used in DLQI calculation in vitiligo patients. DLQI: Dermatology Life Quality Index
